# The start of caring for an elderly dependent family member: a qualitative metasynthesis

**DOI:** 10.1186/s12877-018-0922-0

**Published:** 2018-09-25

**Authors:** Lourdes Moral-Fernández, Antonio Frías-Osuna, Sara Moreno-Cámara, Pedro A. Palomino-Moral, Rafael Del-Pino-Casado

**Affiliations:** Department of Nursing, School of Health Sciences, University of Jaén, Campus Las Lagunillas, s/n, 23071 Jaén, Spain

**Keywords:** Family caregiver, Qualitative, Metasynthesis, Initial, Elderly dependent

## Abstract

**Background:**

The family often takes care of an elderly person who suddenly becomes dependent. This greatly affects different aspects of the caregivers’ lives. The aim of this study is to explore the initial experiences, during the first year of care, of persons who suddenly become caregivers for elderly dependent relatives.

**Methods:**

A search in CINAHL, PsycINFO, WOS, Medline, and Scopus and a metasynthesis of qualitative research were conducted including 19 articles.

**Results:**

Three categories were developed to explain the process of becoming a caregiver ‘taking on the role’ (life changes, uncertainty and confusion, and acceptance or resistance); ‘beginning to realise’ (new needs, impact, and appraisal); and ‘implementing strategies’ (seeking help and self-learning, reordering family and social relationships, solving problems, and devising strategies to decrease negative emotions and stress).

**Conclusions:**

The synthesis provides a comprehensive understanding of the experience of becoming a caregiver in order to help health-care professionals to adapt care plans to this situation.

## Background

The world population has been experiencing a growth in the numbers of older people since the mid-twentieth century, a trend which will no doubt continue through the twenty-first century [1]. This increase in life expectancy is one of the greatest human achievements of recent times, but the fact of living longer carries the likelihood of suffering chronic and disabling diseases that involve increasing dependency [1].

At present, public policy offers low levels of formal long-term care provisions in most EU countries [2, 3]. As a result, family and friends—and especially women—are mitigating this shortcoming. Thus, the figure of the family caregivers appears, as ‘a non-professional person who provides primary assistance with activities of daily living, either in part or in whole, towards a dependent person in his/her immediate circle’ [2].

Taking care of a dependent person involves changes and assumption of new responsibilities by the caregivers, which change their lives significantly. They have to deal with activities of daily living that they previously did not have had to do [4]. Thus, many aspects of caregivers’ physical and mental health, as well as their social and family lives are affected negatively by their caring role [5], causing them problems with their physical health, relationships, personal well-being, and finances [6, 7].

The pathologies that generate dependency can appear progressively or suddenly. The type of appearance of the illness affects the adaptation process of the caregivers [8]. Progressive appearance diseases make the adaptation process easier because of the smooth and gradual onset of the disease. Abrupt or acute appearance diseases require an immediate adoption of the task of caring, and thus, these bring a more sudden change for the caregivers and make the adaptation process more difficult [9]. In this adaptation process, several aspects are involved, among which are the needs, expectations, consequences, and feelings of family caregivers [8].

Research shows that the initial stage of caregiving is the worst, especially when the relative has become suddenly dependent [10].

Helping caregivers to adapt to their role may diminish the negative consequences of care [11, 12]. In order to help this adaptation by implementing the right caregivers’ interventions, it is essential to know the process and characteristics that surround this change.

There are few studies that attempt to clarify the phases that a person goes through as a family caregiver at the beginning of her or his caregiver role, and those that do involve either theoretical research [13, 14] or they focus on specific issues and specific areas [15–18].

Thus, we have perceived a need for a reinterpretation of the whole process of suddenly taking on a caregiver role. The knowledge and in-depth and holistic understanding of the caregivers’ experiences in the early stages will allow us to understand and identify the elements and dimensions involved in the process of becoming a family caregiver. With this, health-care professionals could be better informed and offer interventions that are better adapted to the situation faced by caregivers at the beginning of their caring role, ensuring the well-being of the caregiver and, therefore, of the care recipient as well.

Because there are several qualitative studies that offer an in-depth analysis of the beginning of the caring role, we decided to conduct a qualitative metasynthesis in order to expand the transferability of the results from this research. The aim of this metasynthesis is to explore the experiences during the early stages of a person becoming the caregiver of an elderly dependent relative who has suddenly experienced an acute illness. The specific aims were to identify qualitative research in this area, and through synthesis of the study findings, describe the characteristics and the process of becoming a caregiver.

## Methods

### Design, setting, and sample

We present a qualitative metasynthesis, following the procedures of Sandelowski and Barroso [19]: (a) literature search, (b) quality assessment, (c) analysis of findings, and (d) synthesis of findings. We examined the study findings using constant comparative analysis, and we coded, compared, and sorted conceptual categories, attempting to interpret each category in relation to each other across studies.

### Literature search

To identify qualitative studies, we formulated a comprehensive search carried out from May 2018 to June 2018. To find all relevant studies, we used a multifaceted search strategy with several search strings without time limit for inclusion of the publications. In spite of the difficulties involved in the search for qualitative studies because of the richness and expressive variety of the titles of qualitative research, different alternative terms were searched for the description of the research subject (transition, new or become), following the recommendations of McKibbon, Wilczynski and Haynes [20] and Evans [21] to maximise sensitivity. MeSH (Medical Subject Headings) terms were also added where possible, in order to encompass the most widely used database search such as Medline (caregiver * or carer *).

The search string used was: (Caregiv* or carer*) AND (new caregiv* or become caregiv* or transition in caregiv* or new carer* or become carer* or (transition and carer*)) AND (((“semi-structured” or semistructured or unstructured or informal or “in-depth” or indepth or “face-to-face” or guide or guides) AND (interview* or discussion* or questionnaire*)) or (“focus group” or “focus groups” or qualitative OR ethnograph* or fieldwork or “field work” or “key informant” or “interviews as topic” or “focus groups” or narration or qualitative research or “personal narratives as topic”)).

The five databases searched were: CINAHL, PsycINFO, WOS, Medline, and Scopus. We manually searched the bibliographies of the selected articles to capture relevant research articles that fell outside the identified search terms utilised, through which four more manuscripts were added.

**Fig. 1:**
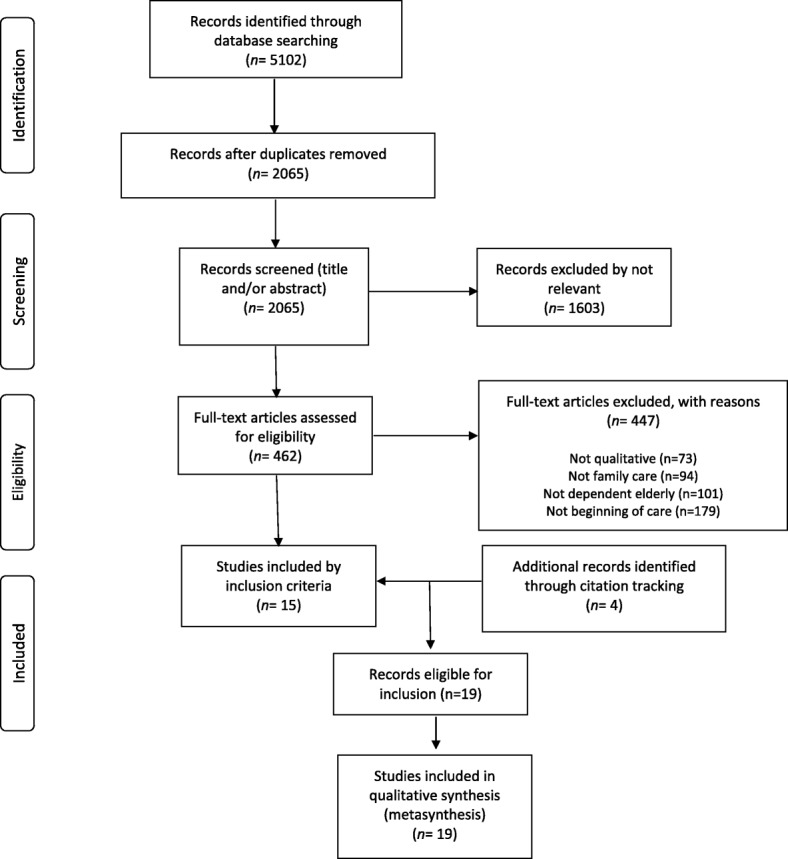
PRISMA(*) flow diagram of the review process. (*) Moher D, Liberati A, Tetzlaff J, Altman DG, The PRISMA Group. The RISMA statement for reporting systematic reviews and meta-analyses of studies that evaluate health care interventions: explanation and elaboration. J Clin Epidemiol. 2009; 62: 1006–1012

The search resulted in a total of 5102 results, of which 3037 were duplicates. Of the 2065 remaining records, 1603 were not relevant to the topic and 462 could fit the inclusion criteria according to the title and/or abstract. The main author reviewed the complete texts of these 462 articles and discarded 447 (73 studies that were not qualitative one, 94 because they did not focus on family care, 101 because they did not study dependent elderly people and 179 because the researches did not occur at the beginning of care). As a result, a total of 15 articles that met the inclusion criteria were selected to be included in the metasynthesis. In addition, four more studies were included by reviewing the references of full texts revised (Fig. 1).

### Study selection

Due to the difficulty of identifying relevant research by keyword searching alone, we manually searched for all the studies which met the inclusion criteria. Papers were reviewed first by title, then by abstract, and finally by full text whenever it was necessary. Inclusion criteria for the metasynthesis were: (a) new primary caregiver (b) caring for less than 1 year (c) for a dependent elderly relative who has suffered an abrupt or acute disease causing dependency; (c) research using qualitative methodology.

### Quality appraisal

According to Sandelowski and Barroso [19], although quality should not be a criterion used to exclude studies, it should be included in the comparative analysis of the design features of each study. For this, we used the Critical Appraisal Skills Programme (CASP) – Qualitative [22], following a prescriptive and comprehensive checklist appropriate for metasynthesis and widely extended in this type of research [23]. The tool consists of 10 questions relating to rigour and relevance, with an assessment system of “yes”, “no” or “can’t tell”. This evaluation of the evidence is based on subjective and judicious discussions about the quality of the recovered evidence. The first two questions (yes / no) are mandatory questions. If the answer is affirmative in both questions, the remaining questions are assessed. The following seven questions are about the research design, data collection and analysis. The last question is about clinical applicability, so we did not use this question.

### Data extraction

After the localisation and selection of the studies, the extraction of the findings from the primary sources was started through manual extraction in each manuscript, including the specific results presented by the researchers as subjects and the dialogues of the participants in the original. This allowed us to reduce the bias of erroneous extraction of data from primary sources [24]. To classify and summarise the studies, the descriptive data of each study were extracted, and the information of each article regarding its design and the characteristics of the sample were recorded in a customised form.

In total, we analysed interview fragments of 393 family caregivers, whose verbatim comments appear in the 19 included articles and are transcribed in 59 pages.

### Data analysis

After the abstraction of data, two of the authors performed the analysis individually, developing categories and coding the data. A dynamic and iterative process of thought, induction, interpretation, theorisation and reflection (chosen against the method of integration and aggregation) were performed. The information was extracted and interpreted, and the findings were obtained through the taxonomic organisation of categories and subcategories, following axial, selective, in vivo, or imported encoding of the same. Everything was done with the help of memos, which arose from the writing of theoretical ideas about codes and their emerging relationships in the coding process [25].

Findings of the research have been introduced in the version 11 NVIVO software, developed by Qualitative Solutions and Research, which allows for the coding of texts in a series of categories that classify dimensions, properties and attributes. The study method and process are presented in detail below.

After a thorough reading of the interview fragments, 693 references resulted and 169 total codes were identified with the highest level of disaggregation. Secondly, we established the characteristics and relationships of the categories and subcategories grouped according to similarities and differences. As a result of this process, and after the consensus of the two researchers involved in the analysis, a total of 16 categories and 10 subcategories emerged.

Finally, a consensus was made between the two researchers involved in the analysis, in order to make the qualitative synthesis, guided by the grounded theory approach [26, 27] through the repeated use of the constant comparative method, for which we looked for similarities and differences in the information available. The memorandums were significant in the process of constant comparison, allowing for the design of the emergency map, and the identification of the concepts and their relationships.

**Fig. 2:**
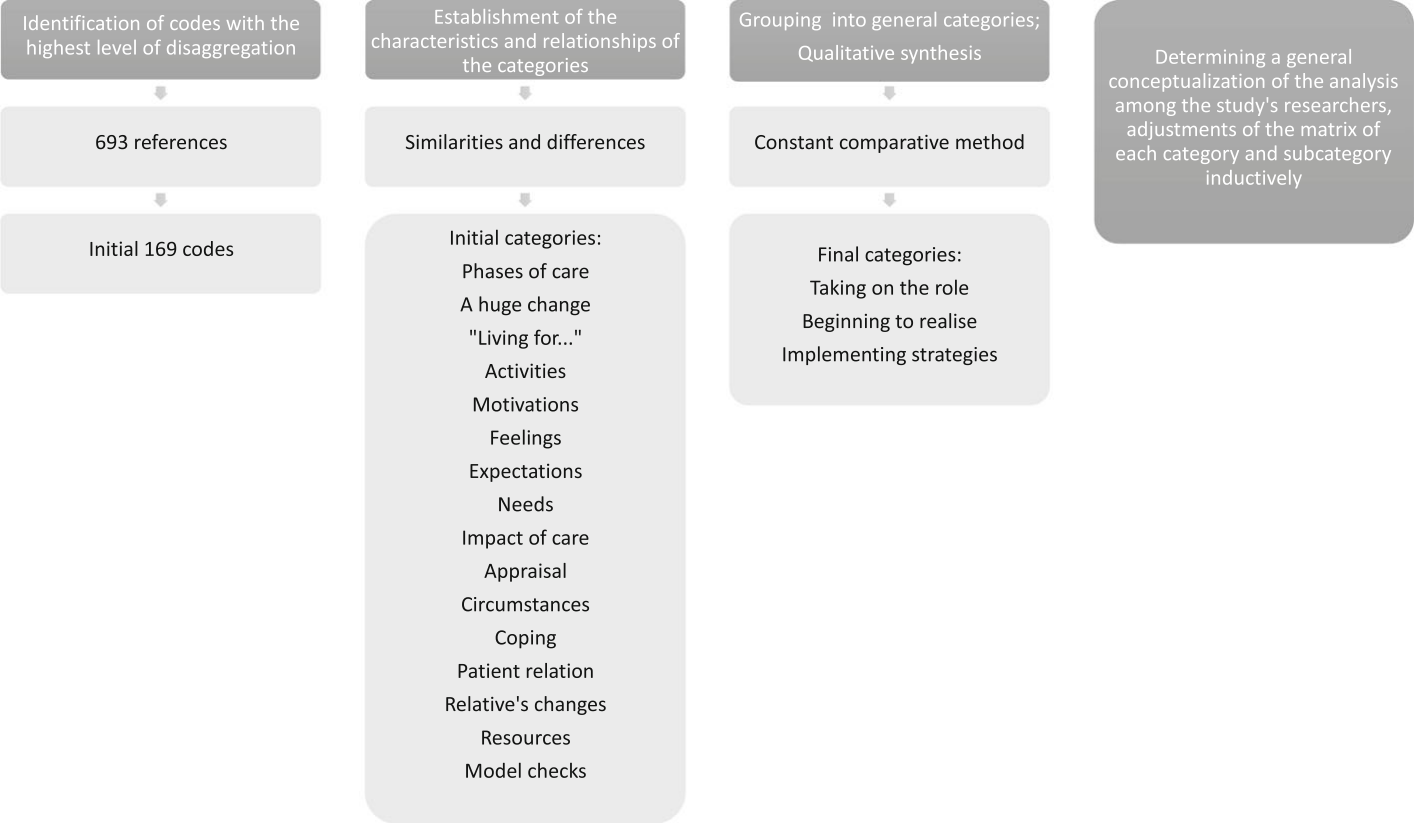
Analysis process

To synthesize the categories, the research team relied on the discourses of the caregivers, repeatedly mentioning the existence of different stages of care. These discourses revealed an initial stage triggered by the illness of his relative and the great change that occurred in all aspects of his life as they began to become caregivers, causing feelings of uncertainty and confusion. From here, caregivers develop different ways of coping, leaving attention, avoiding facing or accepting it, beginning to realize this way of their new role as caregiver. During this phase, caregivers manifest new needs, different consequences of care and re-evaluate their situation with the intention of normalizing it. At this time, the caregivers implement strategies according to their resources, the relationship with the dependent person, the way in which they face their role as caregivers and the circumstances that surround them. Thus, to synthesize the findings, a process of becoming a caregiver with different moments and phases of care was proposed (Fig. 2).

In the last phase of the metasynthesis process, we determine a general conceptualization of the analysis among the researchers of the study, the adjustments of the matrix of each category, and the subcategory and the groups in an inductive manner. The findings were reduced in the coding process, regrouped into ten subcategories integrated into broader categories. This process was carried out according to the relationships of the axis and the care phases appeared in the caregivers’ discourses, and finally three final categories were obtained. To complete the qualitative synthesis, the topics were discussed in several categories by the entire group and a diagram was made with the results of the process of becoming a family caregiver of an elderly dependent.

### Validity

As suggested by Sandelowski and Barroso [19] or Maxwell [28], several procedures can be employed to improve the descriptive, interpretive and theoretical validity of qualitative research integrations.

The descriptive validity was maintained through a thorough search of research related to the subject of study and description of the information of each article included in this metasynthesis, as well as by a CASP quality score assigned by the research team.

Interpretive validity was maintained by team members who analysed the results of the individual studies and integrated them into consensus. In addition, notes were taken during meetings of the research team to maintain an audit trail in decision-making.

The theoretical validity was related to the theoretical constructs that were developed during the study, beyond the description and the interpretation of the primary studies.

## Results

The 19 articles included a total of 393 family caregivers. Most caregivers were women (77%), usually wives and daughters, of various nationalities and with an average age of approximately 50 years. The people cared for were dependent elderly persons (average age of 72 years), with different types of health conditions: stroke, cancer, hip fracture, brain injury, accident, sepsis and transplant.

Of the 19 articles (Table 1), one was written in Portuguese but had translations in English and Spanish, one was written in Spanish, and the others were all written in English. Geographically, five of the articles were from the United States, one from Canada (Ontario), two from South America (Brazil), seven from Europe (Italy, Scotland, England, Spain and Portugal), two from Asia (China), and two from Australia.

**Table 1:** Summary of articles included in the metasynthesis

Author (year), country	Purposes	Methods and sample	Main Results
Houldin (2007) [30] Philadelphia (EEUU)	To report on a descriptive study of patients newly diagnosed with advanced colorectal cancer.	Semi-structured interviews to 14 carers and thematic content analysis.	Findings revealed three domains: Experiencing total disruption of my life, Staying positive, and Attempting to keep family and children’s routines as normal as possible.
Paiva et al. (2012) [31] Río de Janeiro (Brasil)	To discuss the relationship between the family and hospital discharge regarding manifestations, attitudes, feelings and practices.	Semi-structured interviews to 9 carers and theory based on data approach.	Findings reflected the categories: “Facing the unexpected,” “Recognizing the need for care after discharge” and “Realizing one’s importance in the recovery of the relative”.
Plank et al. (2012) [32] Verona (Italy)	To explore and understand the experience of new informal carers in Italy during the transition from hospital to home.	In-depth interviews and focus groups to 18 carers and a qualitative phenomenological approach.	Family caregivers reflected their newly acquired role; the recipient’s condition; and the support they required. The core concept of ‘being responsible for everything’ seemed to be a recurring theme running through these three subject matters.
Grimmer et al. (2004) [36] Australia	To describe the perceptions of people taking on a new or expanded caring role of an elderly patient recently hospitalised with a new or intensified health problem.	Semi-structured interviews to 24 carers (method used not specified).	The study highlighted carers’ perceptions of being unprepared for their new tasks, and their frustrations at the long-term and frequently significant changes to their lives brought about by assuming a caring role.
Teschendorf et al. (2007) [41] EEUU	To describe informal cancer care provision from the perspective of the carer.	Focus groups to 63 carers and content analysis.	Providing supportive care introduced a balancing act in caregivers’ lives as they attempted to address complex and overlapping roles. They felt alone in their decisions, were under-prepared for tasks they assumed, and tried to shield the care recipient
Nahm et al. (2010) [37] Baltimore (EEUU)	To explore informal carers’ experiences with providing care to older adults over the first 6-month trajectory of hip fracture recovery and their support needs.	In-depth interviews to 10 carers and phenomenological approach.	Management of hospital bills and transitions between care settings were especially burdensome. The caregiving situation was viewed as an opportunity to spend more time with their loved ones. Findings revealed unmet support needs expressed by caregivers of older adult hip fracture patients.
Smith et al. (2004) [35] Glasgow (Scotland)	This paper reports on a study that aimed to describe the experience of caring for a stroke survivor at one year after stroke in Scotland.	Semi-structured interviews to 90 carers and thematic data.	Initially most carers found that they lacked the knowledge and skills to care for the stroke survivor at home and so they had to learn how to obtain the information and assistance required. Carers had to adapt to the changes that stroke effected in the stroke survivor and seek alternative ways of securing the resources they needed for managing their lives.
Pereira and Rebelo-Botelho (2011) [38] Portugal	The purpose of this qualitative study was to understand the lived experience of individuals taking on the role of informal adult carers after an unexpected event involving a relative.	Unstructured interviews to 14 carers and Van Manen’s approach.	Four main themes were identified: losing control over time, feeling alone, failing expectations and taking over someone else’s life.
Greenwood et al. (2009) [40] London (England)	To investigate the experiences of informal carers of stroke survivors over time.	Audio-taped in-depth interviews to 31 carers (method used not specified).	A central theme of uncertainty with a number of other interconnected themes were identified. Other themes including adopting routines and strategies, absolute and relative positives and questioning the future could be seen to both influence and be influenced by uncertainty.
Bakas et al. (2002) [39] Oklahoma (EEUU)	To determinate the self-reported needs, concerns, strategies, and advice of stroke carers during the first 6 months after hospital discharge.	Open questions by telephone to 14 carers (method used not specified).	Findings revealed five major categories of caregivers needs and concerns: information, emotions and behaviours, physical care, instrumental care, and personal responses to caregiving.
Weng et al. (2011) [42] Taiwan (China)	To explore the stress experienced by the primary family carer of the living-related liver transplantation patient during the postoperative stage.	Face-to-face semi-structured interviews to 6 carers and thematic content analysis.	Participant stress was caused by the gap between expectations and primary caregiving experiences. In particular, the five themes that were identified: unstable sentiment towards liver transplantation; entanglement of burden; non-synchronized family interaction; distance from the healthcare professional; and concern about the protector role function.
Silva-Smith (2007) [43] Colorado (EEUU)	To describe the process associated with preparing for and beginning a new caring role following a family member’s stroke.	Audio-taped interviews to 12 carers and grounded theory analysis.	Restructuring life for caregiving was associated with five dimensions: daily life, managing multiple roles, relationship with the stroke survivor, future hopes and plans, and time for self.
Brereton and Nolan (2002) [45] Sheffield (England)	To gain a better appreciation of the needs of new carers of stroke survivors and to consider how these needs change as their role develops.	Interviews to 14 carers and grounded theory analysis.	During the initial period carers engage in a number of different ‘seeking’ activities in order to try and ensure that they feel competent, confident and safe to provide care and that they understand the likely future demands they may face. Rather than being facilitated by staff, carers’ efforts often go unnoticed or are overlooked, resulting in carers feeling that they are ‘going it alone’.
Shyu (2000) [46] Taiwan (China)	This study explored the needs of family carers during the transition from hospital to home.	Face-to-face interviews to 16 carers and constant comparative analysis.	Role tuning was the process used by caregivers and care receivers to achieve a harmonious pattern of caregiving and care receiving during the transition from hospital to home.
Shaw et al. (2013) [33] Sidney (Australia)	To explore the experiences of family carers of people diagnosed with upper GI cancer after surgical intervention to (1) identify their unmet supportive care needs and (2) investigate how family carers perceive their role during this time.	Semi-structured telephone interviews to 15 carers and constant comparative analysis.	Family caregivers reported significant information and support needs, their distress was exacerbated by a lack of patient care knowledge. Access to support was limited by caregivers’ lack of understanding of the health system and they view their role as part of their family responsibility.
Rodrigues et al. (2013) [44] Sao Paulo (Brasil)	To examine the transition of care in families caring for elderly persons who suffered the first episode of a cerebrovascular accident.	Interviews to 10 carers and instrumental ethnographic approach.	They describe the care process for the dependent elderly person, strategies for the care process and impact and acceptance of the limitations.
Abrahamson et al. (2017) [34] England	To explore the experiences of individuals who have had a severe traumatic brain injury (TBI) and their carers in the first month post-discharge from in-patient rehabilitation into living in the community.	Semi-structured interviews to 10 patients and nine carers, and thematic analysis.	Many patients and carers felt unsupported in the inpatient phase, during transitions; they struggled to accept a new reality of changed abilities, loss of roles and loss of autonomy, and early experiences post-discharge exacerbated fears for the future.
Giosa et al. (2014) [47] Ontario (Canada)	To explore informal family caregiver experiences in supporting care transitions between hospital and home for medically complex older adults.	Semi-structured interviews to 12 caregivers and grounded theory analysis.	They describe the theory “building capacity to care”, which involves assessing a caregiver’s unique family situation to tailor and provide relevant information, education, and training.
Moral-Fernández et al. (2018) [29] Jaén (Spain)	To describe the initial process through which people who imminently become caregivers of a dependent elderly relative.	In-depth interviews to 11 caregivers, and grounded theory analysis.	An initial phase of changes, in which the caregiver assumes new activities; a second phase full of emotions, in which the needs and consequences emerge; and a third phase that emphasises acceptance as a coping strategy and uncertainty as an expectation of the future.

The different methodological approaches found were: (a) phenomenological design (three works); (b) content analysis (one); (c) thematic content (three); (d) ethnographic design (one); (e) grounded theory (four); (f) constant comparative analyses (two); (g) theory based on data (one); (h) Van Manen’s approach (one); and (i) method used not specified (three). The methods of collecting the information have been interviews and focus groups.

Regarding the methodological quality of the included studies, we found some weaknesses in the following questions: justification of the design used, adjustment of data collection method with the objective and methodology, and the analysis of authors’ role in the research (Table 2).

**Table 2:** Critical Appraisal Skills Program

	Answer 1	Answer 2	Answer 3	Answer 4	Answer 5	Answer 6	Answer 7	Answer 8	Answer 9
Houldin (2007) [30]	Yes	Yes	No	?	?	No	Yes	?	Yes
Paiva et al. (2012) [31]	Yes	Yes	No	Yes	?	No	Yes	Yes	Yes
Plank et al. (2012) [32]	Yes	Yes	Yes	Yes	Yes	?	Yes	Yes	Yes
Grimmer et al. (2004) [36]	Yes	Yes	No	No	?	No	Yes	No	Yes
Teschendorf et al. (2007) [41]	Yes	Yes	No	Yes	?	No	?	?	?
Shaw et al. (2013) [33]	Yes	Yes	No	Yes	?	?	Yes	?	Yes
Smith et al. (2004) [35]	Yes	Yes	No	Yes	Yes	?	Yes	Yes	Yes
Greenwood et al. (2009) [40]	Yes	Yes	No	No	?	No	Yes	Yes	Yes
Bakas et al. (2002) [39]	Yes	Yes	No	?	?	No	Yes	?	Yes
Weng et al. (2011) [42]	Yes	Yes	No	?	Yes	?	Yes	?	Yes
Silva-Smith (2007) [43]	Yes	Yes	No	?	?	No	Yes	Yes	?
Brereton and Nolan (2002) [45]	Yes	Yes	Yes	Yes	?	No	Yes	Yes	Yes
Shyu (2000) [46]	Yes	Yes	No	Yes	Yes	?	Yes	Yes	Yes
Rodrigues et al. (2013) [44]	Yes	Yes	?	Yes	?	No	Yes	No	Yes
Pereira and Rebelo-Botelho (2011) [38]	Yes	Yes	?	Yes	Yes	?	Yes	?	Yes
Nahm et al. (2010) [37]	Yes	Yes	No	?	Yes	No	Yes	No	?
Abrahamson (2016)	Yes	Yes	Yes	Yes	Yes	No	Yes	Yes	Yes
Giosa et al. (2014) [47]	Yes	Yes	Yes	Yes	Yes	No	Yes	Yes	Yes
Moral-Fernández et al. (2018) [29]	Yes	Yes	No	Yes	Yes	No	Yes	Yes	Yes
Yes	19	19	4	12	9	0	18	10	16
No +?	0	0	15	7	10	19	1	8	3
No	0	0	13	2	0	13	0	3	0
?	0	0	2	5	10	6	1	5	3

First, the appearance of different moments or stages of care in the caregivers’ discourses helped the researchers to classify the different categories around different stages of care: *“It has different stages, they change and each stage has its good and bad things. You have to adapt as you can to each situation”* [29].

**Fig. 3:**
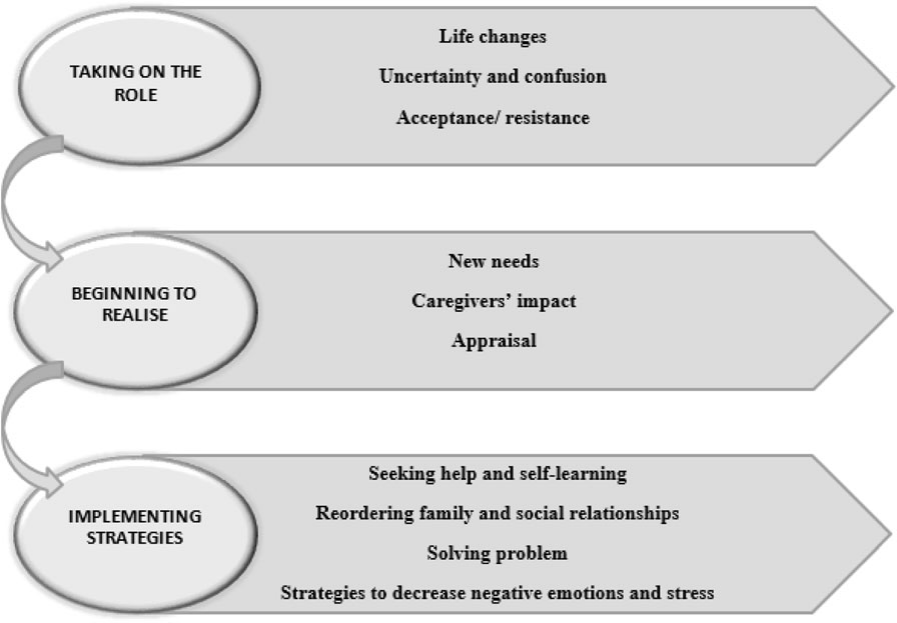
Diagram of the process of becoming suddenly a caregiver of a dependent older relative

According to this fact, and following the analysis, three categories appeared that involved the process of becoming a caregiver: ‘Taking on the role’ (including life changes, caregivers’ uncertainty and confusion, and caregivers’ acceptance or resistance); ‘beginning to realise’ (including caregivers’ new needs, impact, and appraisal), and ‘implementing strategies’ (including seeking help and self-learning, reordering family and social relationships, solving problems, and devising strategies to decrease negative emotions and stress) (Fig. 3).

### Taking on the role

Caregivers feel an initial shock associated with the illness and disability of their family members. Their lives are turned upside down due to the changes associated with becoming a caregiver [29–31].

They mention the new situation that they have to assume and describe the day of onset of their family member’s illness as crucial to their lives and as unpredictable [32–34].

The transition in moving from the hospital to the home tends to be harder than they expected, as it contrasts with the joy they experience after discharge and being in their usual environment, where they find instead a concern and lack of preparation to provide necessary care to their family member [34–36].

### Life changes

The changes in the lives of caregivers when they face an imminent situation of dependence of a family member are very large, since in most cases the dependent person has hitherto led an autonomous life and now, due to an illness or acute problem, suddenly finds him- or herself in a situation of dependence [29, 36–38]. *‘This cancer has been a total disruption of my life. Your whole life changes in 24 hours.’* [30].

The caregiver must then leave his or her life aside and almost exclusively care for their relative. Normally, as the carers exercise their role, their daily activities are multiplied. As a result, caregivers have to cope with a huge amount of new responsibilities related to care.

### Uncertainty and confusion

The changes and the assumption of new responsibilities of care cause diverse emotions for the caregivers, among which the following stand out: the uncertainty, the imprecision of the prognosis related to the illness, for the future that they have to face, and for the lack of information and preparation for the situation [32, 34, 37–41]. *‘They just don’t know—that is what is quite scary, it is the unknown. You just don’t know what it is going to be like in a year’s time… because… everybody is just so different.’* [40].

Although uncertainty is present both in the present and in the future, family caregivers experience different feelings such as: concern about the care situation they must assume; suffering from seeing the discomfort of the family member they are caring for and the change they have undergone; impotence for not being able to do more or take better care of them; or guilt for failing to perform this task as they would consider appropriate or even for wanting to end that situation in any way [29, 32, 33, 38, 41].

### Acceptance or resistance

Given all the problems and needs that family caregivers have, they show different ways of tackling the changes that occur when they begin caring for their relatives. Among these ways that can be adopted of tackling the issue, we find there is generally acceptance of or resistance to the care situation [31–33, 35, 40, 41].

However, other family caregivers are not located on either end, and they underline the lack of knowledge about how to deal with this situation [35]. *‘You have to put up a barrier. I sort of just try to look at it as if it’s not my husband. … Learning to separate yourself from the patient. You get so emotionally involved.’* [41].

### Beginning to realise

Following the assumption of the role and the changes produced, and once in the home, family caregivers begin to realise what the care of their relative entails for their own lives. They begin to be aware of the situation of dependency of their family member, which they sometimes compare with young children, and the amount of care they require for, where possible, an improvement to occur [31, 32].

### Carers’ impact

With the development of caregiving, family caregivers begin to realise the difficulty of this task and the responsibility for be the well-being of their relative being in their hands. Many areas of their lives are altered as a result of their work as a caregiver, starting with their own personal life [33, 38]. *‘We lost our personal life… most of it depends on my mother’s schedule. For us, being still young, it’s hard’* [38].

The performance of so many activities, with the reconciliation of their lives up to the present, presents a challenge to them, causing them stress, weight, and anxiety [29, 32, 37–39, 42]. *‘I have been diagnosed with anxiety. I am nervous and get angry easily. The stress comes from inside myself and causes me to feel tremendous tension. This is a difficult time for me’* [42].

The difficulty in combining so many activities is also due to a lack of time. Continuous supervision of a relative is a great time constraint [29, 35, 43].

As a result of the development of care and restrictions of their time, some family caregivers have repercussions in their working life, and even have to leave their paid work in order to dedicate themselves to the care of their family member: *‘End up abandoning my job, because there was nobody to care for my mother’* [44]. This, together with the economic impact of the development of an elderly person in a situation of dependency, also causes their economic situation to be negatively impacted [29, 35, 39–41, 43].

They do not have time for themselves, they cannot do the same activities they used to do, and even their sleep is affected, either because of concerns about the care situation and their family, or the person’s needs for attention [29, 30, 32, 37, 40]. *‘I’m sleeping less and less, because of all the problems regarding appropriate home environment’* [32].

This lack of sleep also affects the physical state of the caregiver, in addition to the mental state, which causes daily fatigue and exhaustion [29, 33, 37, 39, 41, 45]. *‘I’m getting tired. ‘Cause I need a holiday…I’m just a bit tired, emotionally drained actually’* [33].

Their social life is largely non-existent because they have to spend so much time taking care of their relative. Moreover, relationships between the dependent family member and the caregiver’s nuclear family are affected in this situation, especially in the early stages, because there is a loss of privacy for the family or couple, which can cause family conflict [29, 30, 38, 39, 43, 45]. *‘He never complained of anything, and suddenly he fell in the bathroom, it was such a fright... this situation had an impact on the family relationships, because of his limitations’* [44].

It is also worth mentioning that there are also discourses in which caregivers mention the personal growth and satisfaction that the care task reports to them, and how it helps them value day-to-day things [29, 40, 41]. *‘You appreciate a lot of things, don’t you when you can’t do all the things you did? So I think you learn to know yourself a lot better… and then you think ‘I can’… and there is a certain satisfaction, isn’t there?’* [40].

### New needs

The synthesis of the findings found in the articles on the first moments in the care of a dependent family shows that the main needs of the initial caregivers are the need for information, time, help, support, and instruction for care.

The need for information about the illness, care and prognosis of the family member, and the training to develop the necessary skills to perform the task of caring in the best possible way, is reflected in the requests of family caregivers to health personnel [29, 30, 32, 33, 35–37, 39–41, 43, 45–47]. *‘We would like to know how ill she is; I think the hospital should give us a card and state what kind of disease the patient has, how well the disease has been treated, and whether there are any risks left, so the family can know what to expect.’* [46].

They emphasise expressions like, ‘there was nobody there that could tell me’, ‘no one tells you anything’, ‘nobody really told me anything’, from which the presence of personnel is glimpsed, without any offer of information or help.

They do not feel sufficiently prepared to take care of their relative with all the problems that the patient may present at any time [34, 41, 42, 47].

Caregivers also complain about the need for financial resources, because caring for a dependent person entails a lot of expenses and they do not normally have a paid job, or as stated in the previous category, they have had to leave a job because of the care [29, 35, 46].

They find it difficult to balance care with their other responsibilities, and for this reason they express that aid is one of the most important needs, whether at the professional level, by health personnel; or family, in many cases no other family member is involved in care [32, 33, 43, 45].

Family caregivers not only do not receive help in caring for their relatives, they do not receive support for themselves. Hence they express the need for someone to go and see them and talk to them to get their act together [29, 38, 46]. *‘I feel like I am in a room with thousands of people and I am screaming out and no one is hearing me.’* [43].

### Appraisal

According to Lazarus and Folkman [48], appraisal is a process by which a person evaluates the situation and makes changes in his or her behaviours or skills needed to cope with the stressful situation. In this case, family caregivers generally feel that they cannot cope with this task properly and need help in their functions and resources to continue in the right way [33, 36, 45]. *‘It really battered me around… it really wears you down. I couldn’t cope I had to get out…I’m tired of it.’* [35].

### Implementing strategies

After assuming the role of caregiver and beginning to realise the consequences, caregivers try to adopt certain strategies that make this task of care more bearable for them.

### Seeking help and self-learning

Among these strategies adopted, caregivers try to learn about the disease that affects their family and the dispensing of care required, thus seeking both formal and family help, and they try to learn care skills [36, 40, 45, 47]. *‘I started reading a lot about what to expect*. *.*. *so I tried to understand what she is going to be changing into’* [40]. “*We were pulling it [looking for information] on our own because otherwise it was just a black hole … you’re kind of thirsting for information that whole time”* [47]*.*

### Reordering family and social relationships

As stated earlier, one of the consequences of taking on the carer role is the changing of their social and family relationships, so that caregivers seek to reorganise these relationships to normalise the situation. Thanks to this family organisation, the work is done as a routine that is divided into jobs for each member of the family, which creates a greater sense of family calm [40, 43]. *‘I think that we’re calming down, I can feel this too, I myself am much calmer! But I still think the insecurity affects us because it’s all very new, you know? However, I feel, like my sisters, that we’re restructuring...it’s because time is passing.’* [43].

### Solving problems

Following trying to normalise the situation of care, caregivers may adopt coping strategies focused on solving problems, which increases safety in the care they provide [29, 38, 40]. *‘I think that every time we get a little bit further away, it makes us more secure. It is like dangerous waters and we are gradually sailing out of them’* [40].

### Strategies to decrease negative emotions and stress

Finally, in the process of becoming a caregiver, they seek to reduce the negative effects by trying to take care of strategies to maintain emotional positivity and reduce stress levels such as talking to the family and letting go, taking things intentionally humorously, being positive and optimistic, and encouraging the person cared for to maintain spiritual support, among others [30, 31, 40]. *‘One thing that – whatever happens, I always say that it is [the] best. Better. God never wants to harm you, it is your destiny. And there is always something positive – good.’* [40].

## Discussion

The findings of the present metasynthesis respond to the objective proposed in the research and show that the process by which people suddenly become caregivers of an elderly relative in a situation of dependence is dynamic and progressive in time. It happens when a family member develops symptoms that make it difficult to carry out the activities of his daily life, and a person from his family environment helps him with the care needs that arise, until this person who takes on the caregiver’s role begins to realise the consequences and tries to normalise his new role of caregiver.

There is no article that indicates the generic phases through which a caregiver passes to take care of a dependent family suffering an acute-onset illness, but we found articles that describe stages of this transition in dementia [15, 17, 18], research about the stages towards cohabitation [16], the process of adaptation to dependency in older adults and their families [49], some theoretical approaches [13], and another review about family care in terminally ill patients [50]. Firbank and Johnson [16], examine the paths that lead elderly people and their family caregivers to cohabiting. Bunn et al. [15] evaluate the qualitative evidence about how people accommodate and adapt to the diagnosis of dementia and its immediate consequences. Prorok et al. [18] try to understand the health-care experience of people with dementia and their carevigers. Gibbons et al. [17] try to understand the experience of family caregivers during the liminality phase. Finally, Martín et al. [50] reveal the experience of family caregivers who care for a terminally ill patient in their home. The results of these studies coincide roughly with our results, so that Firbank and Johnson [16], Bunn et al. [15], Gibbons et al. [17], and Martín et al. [50] first identified a health event that causes some kind of dependency in a family, which means that a family member takes over care, hence after diagnosis, the stage of change begins.

A common event in all phases of research dedicated to the process of becoming a caregiver is the changes. This research shows that the trigger with which the process of becoming a caregiver begins is the great change caused by a dependence situation in a family member. This is widely supported with various studies. Bunn et al. [15] and Moreno-Cámara et al. [11] describe these changes in a way that is similar the present research; during the first phase of care, when the family has been diagnosed with dementia and begins with a succession of changes that interfere with all areas of the lives of the caregiver, and to which they must adapt. Gibbons et al. [17], speaks about a pivotal event (disability or illness) in the pre-liminal phase that turns the lives of caregivers upside down and causes changes in the intermediate stage of care; in particular they highlight social changes. Prorok et al. [18] agree with our results that identify confusion and uncertainty early in the process, and identify changes in the last phase of the start of care, in which the caregiver must live with these changes. Changes are also a constant in most of the research on the early stages of care [9, 50–55]. In spite of ‘change’ being a category as such individually, it is also the precursory of other categories and influences in the development of them, not only because of its meaning but because all other categories that make up the process of becoming a caregiver happen with subsequent changes. For this reason, it is a dimension identified in the early stages of care.

In this first phase of assuming the role of caregiver, a range of emotions experienced by carers will accompany the changes, highlighting the uncertainty and suffering that coincide with those expressed by Gibbons et al. [17] in the liminal and post-liminal phase in their research about the beginning of caring, and by Moreno et al. [11]. These emotions have also consistently surfaced in several original studies and reviews about the beginning stage of care [54, 56] and they are widely developed in the studies of this metasynthesis, from comforting and satisfying emotions to emotions of fear and despair.

In the second stage, caregivers begin to realise their new role. In this way, new needs arise that are associated with the care of their relative, and they start to experience the consequences of exercising this role. The results of this metasynthesis resemble those found in Prorok’s article [18], which identifies the need for information during the early stages of care, Bunn’s article [15], which also describes the second phase of the start of care as a time when conflicts arise, and Firbank’s research [16], which explains the elderly person’s inability to perform activities of daily living and the children’s needs in providing help to their parent. Also, the needs that emerge in the early stages of caring are matched in studies of caregivers of people who have had a stroke [56, 57], and the consequences coincide, to a large degree, with the results most commonly found in other research into the early stages of care [55, 58, 59].

During the third phase, which involves implementation of strategies to minimise the negative effects of care, the emotional consequences and stress occur. Caregivers seek to learn the skills to become more self-sufficient in care and finally reorganise relations for a better adaptation of both the family and social situation. Among these implemented strategies we can find coping strategies, which include seeking help and solving the problems that arise during care. The latest strategy is, according to Gottlieb and Wolfe [60], the most important and better coping strategy. This coincides roughly with the third stage proposed by Bunn et al. [15] where, as in this metasynthesis, the implementation of strategies that help to minimise the impact of care was located after the initial changes, and with Prorok’s [18] and Moreno-Cámara’s research [11], identifying self-learning as also being an important strategy of implementation. All these facts mark a new situation of normality for the family caregiver [17, 48], thus showing a sample of acceptance and normalization of her new role and culminating the process of conversion to caregiver.

It is interesting how significant differences were not found in relation to the different pathologies that the dependent person suffered, but to their level of dependence and the abilities of the caregiver to develop adequate coping strategies. The phases proposed in this research can be related to the common phases of a crisis, referred to as ‘a temporary event with a start and an end that appears unexpectedly and whose course follows the following phases: impact phase, reaction phase and phase of reorientation’ ([61] p. 51). In agreement with the results of the present research and the phases proposed by Ruiz [61], it could be determined that the people who become caregivers at first are impacted due to the unexpected character of their new role, stunning him and affecting him emotionally. This could correspond to a state of shock, which is characterised by a state of shock and blockage. The reaction phase is related to the awareness of the situation, which implies suffering and different emotional reactions. After that, the reorientation phase is characterised by the assimilation of the situation, whereby the caregiver begins to accept what has happened and is mobilised to face and overcome the situation, producing in this way a readjustment in their lives in search of a new stability.

The results of this metasynthesis help us to understand how a relative takes care of a dependent elderly person and how this care evolves until the caregiver begins to adapt to this role. Knowing the stages of the process of becoming a caregiver may be useful for health professionals in establishing interventions tailored to each stage in order to help caregivers to adapt to their new role and resolve their uncertainty and inexperience.

Because the first moments of care have a common succession of changes triggered after diagnosis, which causes feelings of confusion, suffering, and uncertainty, early interventions might be appropriate based on generic information about the disease that has led to the dependence of the older person, the care needs during the first moments of care, and information about available resources, in order to alleviate feelings of confusion and uncertainty experienced by caregivers. During the second and third stages, caregivers evaluate the caring situation and implement strategies to adapt to the caring situation; at this time, tailored interventions are needed to help caregivers in assuming their role, implementing better adaptive strategies, and solving care needs.

### Limitations

The main limitation of this research is the lack of availability of the complete original transcripts of the primary studies, which hinders and influences the researchers’ analysis of this metasynthesis. This research is based on fragments of caregiver discourses that the primary authors have selected as a reflection of their results. To try to solve this limitation, two different researchers have analyzed the discourses independently and unrelated to the interpretation of the original authors.

With respect to the quality of the studies included in metasynthesis, a good quality of the included studies has generally been observed. According to the CASP tool, a large number of responses are positive, although criteria related to the justification of the research design in relation to the aims of the research, the explanation of the data collection and the consideration of the relationships between the researcher and the study participants. Although the first two criteria are evaluated in a deficient manner, it is possible to observe the congruence of designs and data collection methods used in the included studies with the analysis of the initial experiences of persons who suddenly become caregivers for elderly dependent relatives, even though these issues are not specifically detailed in most of the articles of the present review. Thus, it would have a minimal impact of the low quality in the previous criteria on the review’s quality. This possible minimal impact is based not only in previous reasoning but also in the assertion of Sandelowski and Barroso, which argued that the quality of the studies should not determine the integrity of the research [19].

### Transferability

The analysis of qualitative information included in this metasynthesis, which comes from different cultural contexts and various caregiver profiles, means these results are useful for understanding the earliest stages of care for dependent elderly family members in different situations.

In addition, both the search strategy and the characteristics of each included study have been described in detail, which allows its replication, and may facilitate transferability to other similar situations.

## Conclusions

The main contribution of this study is the identification of the process characterised through the stages of ‘taking on the role’, ‘beginning to realise’, and ‘implementing strategies’. At the beginning stage of caring for a dependent family member, it is usual for the caregiver to experience changes that affect most aspects of the caregiver’s life, such as the responsibilities they assume and the activities they must perform once they become carers. As they have to perform all the activities and meet all the needs that the patient cannot do for themselves, there is an increase in their own needs that is provoked by the new situation that they have to deal with regarding their caring role. All this entails some consequences for the caregiver, which often tend to be emotional, familial, and social. Finally, the carer of an elderly person who becomes dependent suddenly seeks strategies to normalise their situation as caregiver and reduce stress.

This study will allow us to learn more about the transition to the role of caregiver in order to propose a more detailed plan of care, with specific interventions during the early days of caring. Early interventions based on generic information may be needed at the beginning of the process in order to alleviate uncertainty, and tailored interventions may be needed later, depending on the evaluation of the caring situation, in order to help caregivers with implementing adaptive strategies.

We recommend that future research should include the development of studies that address the process of becoming a primary caregiver as a phenomenon of global study with primary data.

## Abbreviations

CASP: Critical appraisal skills programme
GI cancer: Gastrointestinal cancer
MeSH: Medical subject headings
TBI: Traumatic brain injury
